# Diagnosis of Diseases of the Brain and Spinal Cord

**Published:** 1886-06

**Authors:** 


### BOOK REVIEWS.

---

DIAGNOSIS OF DISEASES OF THE BRAIN AND OF THE SPINAL CORD. *By* W. R. GOWERS, M. D., F. R. C. P., ASSISTANT PROFESSOR OF CLINICAL MEDICINE IN UNIVERSITY COLLEGE; PHYSICIAN TO UNIVERSITY COLLEGE HOSPITAL AND TO THE NATIONAL HOSPITAL FOR THE PARALYZED AND EPILEPTIC. *Pp. VIII, 293. New York:* WILLIAM WOOD & CO. *Chicago:* W. T. KEENER.

This work bears the same relation to the general subject of the diseases of the nervous system that one on physical diagnosis does to internal medicine. The object is to give the student a clear idea of the general principles upon which a diagnosis is made, and only so much of anatomy and physiology as will enable him to come to a conclusion.

The first eighteen lectures are devoted to the study of the brain and its disorders and are published in response to a request from many readers of the author's work on the "Diagnosis of the Diseases of the Spinal Cord." Those who have formed a high opinion of Professor Gowers' work will not be disappointed by a perusal of this work on the brain. He brings to the task the same ripe clinical experience, thorough knowledge of all the latest researches in this special branch, and lastly a keen appreciation of the difficulties which beset the beginner.

The last portion of the book is merely a reprint of the author's work on the cord. It is not mentioned in the preface, and is evidently merely bound in the volume by the publishers. This arrangement leads to a certain want of continuity which the reader will find does not exist when studying these disorders.

We trust the author will remedy this defect by incorporating these two excellent monographs in a larger work on the "Diagnosis of Diseases of the Nervous System."

